# Dr. R. G. Snow

**Published:** 1871-08

**Authors:** 


### OBITUARY NOTICE.

Dr. R. G. Snow, well known to the dental profession throughout the country, from his connection with the "Snow & Lewis Automatic Plugger, etc. etc.," died in Buffalo, N. Y., in May last of typhoid fever.

Dr. Snow was born in Madison County, New York, December 28th, 1805, and was, consequently, in his 66th year . His par-removed to Chautauqua County during his early years, whence he came in the spring of 1827 to Buffalo, and spent some time in teaching. He afterwards entered the office of Drs. Chapin and Burwell as a medical student, and finished the prescribed course of instruction by receiving the degree of M.D. from the Fairfield Medical College, in the spring of 1833.

He commenced the practice of medicine in Sandusky city, Ohio, where he remained two years. Subsequently he returned to Buffalo, and practiced his profession in partnership with Dr. Bryant Burnwell. In 1839 he turned his attention to dentistry, as his health would no longer permit him to undergo the fatigue and exposure consequent upon following the general practice of medicine. In this branch of the healing art he received instructions from Dr. Uriah Dunning, who was then a prominent dentist in this city, and with whom he eventually formed a partnership. Dr. Dunning soon after left the city, and Dr. Snow then resumed the practice connected with the office, which was continued throughout a period of nearly thirty years, without any lengthened interruption.

Dr. Snow was a man of conceded ability in his profession, and took a very active interest in its progress and welfare. He was one of the first dentists in the part of the State where he resided, and stood foremost in all movements looking towards the improvement of dentistry.

He was once President of the Western New York Dental Association, and under the present State Law was also appointed as Censor from the Eighth District Dental Society, which position he occupied at the time of his death, and was not inferior in general intelligence and attainments to any member of the Board.

